# Distinctive Regulation of Emotional Behaviors and Fear-Related Gene Expression Responses in Two Extended Amygdala Subnuclei With Similar Molecular Profiles

**DOI:** 10.3389/fnmol.2021.741895

**Published:** 2021-09-03

**Authors:** Shuhei Ueda, Masahito Hosokawa, Koji Arikawa, Kiyofumi Takahashi, Mao Fujiwara, Manami Kakita, Taro Fukada, Hiroaki Koyama, Shin-ichiro Horigane, Keiichi Itoi, Masaki Kakeyama, Hiroko Matsunaga, Haruko Takeyama, Haruhiko Bito, Sayaka Takemoto-Kimura

**Affiliations:** ^1^Department of Neuroscience I, Research Institute of Environmental Medicine, Nagoya University, Nagoya, Japan; ^2^Molecular/Cellular Neuroscience, Nagoya University Graduate School of Medicine, Nagoya University, Nagoya, Japan; ^3^Research Organization for Nano and Life Innovation, Waseda University, Tokyo, Japan; ^4^Department of Life Science and Medical Bioscience, Waseda University, Tokyo, Japan; ^5^Precursory Research for Embryonic Science and Technology, Japan Science and Technology Agency, Tokyo, Japan; ^6^Laboratory for Systems Neurosciences and Preventive Medicine, Faculty of Human Sciences, Waseda University, Tokorozawa, Japan; ^7^Research Institute for Environmental Medical Sciences, Waseda University, Tokorozawa, Japan; ^8^Department of Neurochemistry, Graduate School of Medicine, The University of Tokyo, Tokyo, Japan; ^9^Department of Nursing, Tohoku Fukushi University, Sendai, Japan; ^10^Computational Bio Big-Data Open Innovation Laboratory, National Institute of Advanced Industrial Science and Technology, Tokyo, Japan; ^11^Institute for Advanced Research of Biosystem Dynamics, Waseda Research Institute for Science and Engineering, Waseda University, Tokyo, Japan

**Keywords:** central extended amygdala, gene expression, fear, anxiety, central nucleus of the amygdala, bed nucleus of the stria terminalis

## Abstract

The central nucleus of the amygdala (CeA) and the lateral division of the bed nucleus of the stria terminalis (BNST) are the two major nuclei of the central extended amygdala that plays essential roles in threat processing, responsible for emotional states such as fear and anxiety. While some studies suggested functional differences between these nuclei, others showed anatomical and neurochemical similarities. Despite their complex subnuclear organization, subnuclei-specific functional impact on behavior and their underlying molecular profiles remain obscure. We here constitutively inhibited neurotransmission of protein kinase C-δ-positive (PKCδ+) neurons—a major cell type of the lateral subdivision of the CeA (CeL) and the oval nucleus of the BNST (BNSTov)—and found striking subnuclei-specific effects on fear- and anxiety-related behaviors, respectively. To obtain molecular clues for this dissociation, we conducted RNA sequencing in subnuclei-targeted micropunch samples. The CeL and the BNSTov displayed similar gene expression profiles at the basal level; however, both displayed differential gene expression when animals were exposed to fear-related stimuli, with a more robust expression change in the CeL. These findings provide novel insights into the molecular makeup and differential engagement of distinct subnuclei of the extended amygdala, critical for regulation of threat processing.

## Introduction

Fear and anxiety are evolutionarily conserved across the species and are essential physiological functions for preservation against threatening stimuli. They are considered distinguishable. Fear is a phasic emotional state induced by identifiable, predictable, and/or imminent threats, while anxiety is a sustained emotional state induced by uncertain, unpredictable, and/or physically distant threats (Davis et al., 2010; Sylvers et al., 2011; Tovote et al., 2015; Knight and Depue, 2019). In the functional domain of negative valence systems, the Research Domain Criteria framework—established by the National Institute of Mental Health—distinguishes “fear” and “anxiety” as an “acute threat” and “potential threat,” respectively. The understanding of these negative valence systems is currently inadequate; therefore, elucidating the molecular, cellular, and circuitry functions in threat processing systems is required for treating psychiatric disorders.

The amygdala and the bed nucleus of the stria terminalis (BNST) are two major brain regions involved in negative valence systems (Davis et al., 2010; Fox et al., 2015; Ahrens et al., 2018; Fox and Shackman, 2019). Both regions have complex subnuclear organizations: the amygdala includes up to 13 nuclei and the BNST includes up to 20 nuclei (Fox et al., 2015). Among them, the central nucleus of the amygdala (CeA) and the lateral division of the BNST (lateral BNST), are extensively reciprocally connected, comprising a functional-anatomical macrosystem known as the central extended amygdala, which is a pivotal brain region for threat processing (Alheid and Heimer, 1988; Alheid et al., 1998; Dong et al., 2001a; Fox et al., 2015). Similarities between the CeA and the lateral BNST at the molecular, cellular, and circuit levels have been discussed (Moga et al., 1989; Shimada et al., 1989; Gray and Magnuson, 1992; Fox et al., 2015; Ye and Veinante, 2019). Several recent human imaging studies have reported that the activity levels of the CeA and the BNST differ depending on threat timing and type (Klumpers et al., 2017; Gorka et al., 2018; Hur et al., 2020). Patients with generalized anxiety disorder showed decreased activity in the amygdala and increased activity in the BNST during gambling games inducing sustained anxiety (Yassa et al., 2012). These human studies suggest that the CeA and the BNST contribute to threat processing in a distinctive manner; however, these studies lacked sufficient spatial resolution to investigate the similarities and differences at the subnucleus level. Rodent studies focusing on the extended amygdala have also been conducted extensively in recent years, which have elucidated the subnuclear circuitry mechanisms of threat processing. However, few studies have focused on the direct comparison between the CeA and the BNST subnuclei at molecular and cellular levels.

In this study, a known genetically identified cell type in the central extended amygdala, PKCδ-positive (PKCδ+) neurons, was focused on to compare subnuclear function and molecular profiles. PKCδ+ neuron is a major cell type of the lateral subdivision of CeA (CeL) and the oval nucleus of the BNST (BNSTov; also known as BSTLD in the atlas; Paxinos and Franklin, 2012). We found that constitutive inhibition of neurotransmission from PKCδ+ neurons in these subnuclei showed double dissociation of their contribution on the regulation of fear and anxiety, respectively; the PKCδ+ neurons in the CeL were essential for fear learning, whereas the neurons in the BNSTov were significantly involved in anxiety expression. In order to reveal subnuclei-specific molecular profiles, PKCδ+ neurons were labeled with a fluorescent protein in a genetically modified mouse brain, and subnuclei-targeted microsamples were obtained by a micropunch-out system to conduct RNA sequencing (RNA-seq). Differential gene expression analysis showed that the CeL and the BNSTov had relatively similar gene expression profiles among subnuclei at the basal level. On the other hand, consistent with their subnuclei-specific engagement in behavior, they displayed distinctive gene expression profiles when animals were exposed to fear-related stimuli; in particular, a more robust change in gene expression was observed in the CeL after fear-conditioning. The findings of the present study provide novel insights into the molecular makeup and differential engagement of distinct subnuclei of the central extended amygdala, critical for regulation of emotional behaviors.

## Materials and Methods

### Mice

All experiments were conducted in accordance with the Nagoya University Regulations on Animal Care and Use in Research and were approved by the Institutional Animal Care and Use Committee, Nagoya University (approval number R210154). Mice were group-housed after weaning and kept under 12-h light/dark cycle with food and water provided *ad libitum*.

*Prkcd-cre* mice {Tg(Prkcd-glc-1/CFP,-cre)EH124Gsat; stock #011559-UCD} were obtained from the Mutant Mouse Resource and Research Center. *Sst* (somatostatin)*-cre* mice {Sst tm2.1(cre)Zjh/J; stock #013044} and Ai14 mice {B6.Cg-Gt(ROSA)26Sor tm14(CAG-tdTomato)Hze/J; stock #007914} were obtained from the Jackson Laboratory. *Crh* (corticotropin-releasing hormone)*-cre* mice {Crh<tm2(cre)Ksak>} were described in the previous study (Itoi et al., 2014). Wild-type C57BL/6J mice were purchased from SLC Japan (Shizuoka, Japan).

### Stereotaxic Viral Injection

AAV1/2-EF1α-DIO-mEGFP-WPRE (6.8 × 10^12^ genomes/ml) and AAV1/2-EF1α-DIO-GFP-TeNT (1.0 × 10^13^ genomes/ml) were prepared as previously described (Kawashima et al., 2013). Mice were anesthetized with chloral hydrate (400 mg/kg, i.p.) and xylazine (2 mg/kg, i.m.), and then fixed in a stereotactic frame (Model 942; Kopf Instruments). The skull surface was exposed via a midline sagittal incision and treated with local anesthetic lidocaine hydrochloride jelly. Small craniotomies were then performed using a microdrill. Glass capillaries were inserted bilaterally into the BNSTov (coordinates from bregma; AP +0.2, ML ± 1.2, DV −3.5, coronal angle 20°) or the CeL (AP −1.35, ML ± 3.0, DV −4.6) according to the atlas (Paxinos and Franklin, 2012). AAV solutions (0.3 or 0.5 μl) were loaded into the BNSTov or the CeL, respectively. *Post hoc* histological analyses were performed after behavioral tests to confirm the bilateral injection of the virus.

### Behavioral Procedure

#### Open Field Test

Each mouse was placed in a corner of the 40 cm × 40 cm × 30 cm light gray open field (OF) apparatus illuminated at 100 lux. Spontaneous locomotor activities were recorded for 10 min, and the total moving distance was calculated using the automated video tracking software TimeOFCR1 (O’Hara & Co., Tokyo, Japan).

#### Elevated Plus Maze Test

The elevated plus maze (EPM) consisted of two opposite open arms (25 cm × 5 cm × 0.3 cm) and two enclosed arms (25 cm × 5 cm × 10 cm), with 5 cm × 5 cm central cross area illuminated at 6 lux and elevated to a height of 50 cm above the floor. Each mouse was placed on the central cross area of the maze. Mouse activities were recorded for 10 min, and the time spent on each arm was analyzed using the automated video tracking software TimeEP1 (O’Hara & Co.). The percentages of time spent in either open or closed arm over total time were calculated.

#### Contextual and Cued Fear Conditioning Tests

The experiments were conducted over the course of three consecutive days. On day 1 for the fear conditioning session, each mouse was placed in a 10 cm × 17 cm × 10 cm shock chamber, which consisted of clear acrylic walls and a metallic grid floor illuminated at 200 lux (chamber A). A 10 kHz–65 dB tone, which served as the conditioned stimulus (CS), was presented for 30 s; during the last 2 s of the CS, a 0.5 mA electrical footshock was delivered, which served as the unconditioned stimulus (US). After 2 min of acclimation period, five CS–US stimuli were presented every minute. For gene expression analysis, only CS stimuli were given to control group mice. Contextual tests were conducted 24 h after conditioning in the chamber A for 5 min. Cued tests were conducted 48 h after conditioning in altered context with a 10 cm × 17 cm × 10 cm white acrylic chamber illuminated at 50 lux (chamber B). After 2 min of acclimation period, CS stimulus was presented for 3 min.

Freezing responses to CSs (for conditioning and cued test) or context (for contextual test) were analyzed using the automated video tracking software TimeFZ2 (O’Hara & Co.).

### Antibodies and Reagents

All antibodies used in this study were purchased commercially: a mouse monoclonal antibody against PKCδ (610398; BD Bioscience) and secondary antibodies conjugated to Alexa Fluor 488 (A11029; Thermo Fisher Scientific). Neuronal cell bodies and nuclei were visualized with NeuroTrace 435/455 blue-fluorescent Nissl stain (N21479; Thermo Fisher Scientific) and Hoechst 33342 (H3570; Thermo Fisher Scientific), respectively.

### Immunohistochemistry

Mice were anesthetized with a combination anesthetic (0.75 mg/kg medetomidine hydrochloride, 4 mg/kg midazolam, and 5 mg/kg butorphanol tartrate) and transcardially perfused with phosphate-buffered saline (PBS), followed by 2% paraformaldehyde in PBS. The isolated brains were postfixed with 2% paraformaldehyde overnight; 30-μm-thick coronal sections were prepared using Cryostat (CM3050S; Leica, Wetzlar, Germany). The sections were washed with PBS, permeabilized with 0.3% Triton X-100 in PBS, and then blocked with blocking solutions [0.3% Triton X-100, 5% normal goat serum (005-000-121; Jackson ImmunoResearch Laboratories), and 1% bovine serum albumin in PBS] for 1 h at room temperature. The sections were then incubated with the diluted primary antibodies in the blocking solution for 3 days at 4°C. After washing with PBS, the sections were incubated with the secondary antibodies and Hoechst 33342 in 0.3% Triton X-100 containing PBS for 1 h at room temperature.

Low-magnification tiling images were acquired using an all-in-one fluorescence microscope BZ-9000 (KEYENCE, Osaka, Japan). Confocal images were acquired using an LSM 710 confocal microscope (Zeiss, Oberkochen, Germany). The images were adjusted and labeled using the ImageJ software (NIH).

### Cell Counting

The regions of interest for cell counting were determined by PKCδ-immunolabeled areas between anteroposterior axis +0.35 and +0.15 mm for the BNSTov and −1.20 and −1.70 mm for the CeL from bregma. The total cell numbers were counted using the ImageJ software by Hoechst 33342 signals. PKCδ+ cells and *Ss*t+/*Crh*+ cells were manually marked by PKCδ-immunolabeled signals and tdTomato signals, respectively. Each cell number and overlapping cell number was counted using the ImageJ software.

### Microtissue Collection for RNA-seq

*Prkcd-cre; Ai14* mice were euthanized by cervical dislocation, and the isolated brains were immediately frozen in crushed powder dry ice. Fresh frozen brains were coronally sliced at a 20-μm-thick using Cryostat (CM3050S; Leica), mounted on polyphenylene sulfide Frame Slide (11600294; Leica), and immediately dried up by air blowing. Microtissue dissection was performed with a punching needle with an inner diameter of 110 μm under a fluorescence microscope as previously described (Yoda et al., 2017). The fluorescence of tdTomato was used as guide markers for punching out PKCδ+ and PKCδ- subnuclei (Figures 3A–C and Supplementary Figure 1B). The sampling positions of all sections were verified using an all-in-one fluorescence microscope BZ-X710 (KEYENCE) before and after punching out. The punched microtissue sections were individually dispensed into the polymerase chain reaction (PCR) tubes filled with 99.5% ethanol and stored at −80°C until RNA-seq library preparation.

**FIGURE 1:**
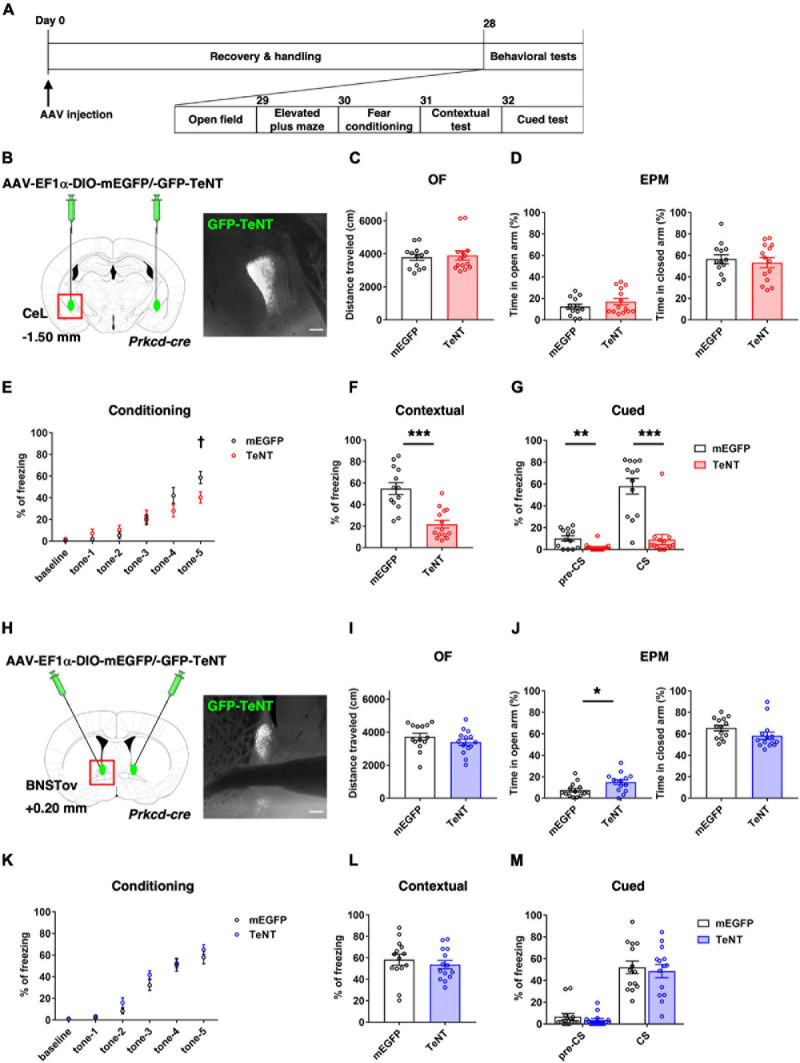
TeNT-mediated synaptic silencing of PKCδ+ neurons. **(A)** Schematic diagram of virus injection and behavioral testing schedule. **(B,H)** Schematic of bilateral injection of cre-dependent TeNT-expressing AAVs into the CeL (**B**, control, *n* = 13; TeNT, *n* = 14) and the BNSTov (**H**, control, *n* = 14; TeNT, *n* = 14), and representative images of the TeNT expression. Scale bars, 200 μm. **(C,I)** Total moving distance during 10 min in the OF {unpaired *t*-test, *p* = 0.749 for **(C)**, *p* = 0.260 for **(I)**}. **(D,J)** Percentage of duration in open arm (left) and closed arm (right) during EPM test {unpaired *t*-test, *p* = 0.240 and *p* = 0.614 for **(D)**, *p = 0.018 and *p* = 0.113 for **(J)**}. **(E,K)** Percentage of time in freezing during fear conditioning of 2 min pre-tone (baseline) and five times of CSs presentation {two-way RM analysis of variance, F_interaction_ [5, 125] = 3.826, **p = 0.003 for **(E)** with Holm–Sidak’s *post hoc* tests, ^†^*p* = 0.026 at tone-5, *F*_interaction_ [5, 130] = 0.707, *p* = 0.619 for **(K)**}. **(F,L)** Percentage of time in freezing during contextual test {unpaired *t*-test, ***p < 0.001 for **(F)**, *p* = 0.485 for **(L)**}. **(G,M)** Percentage of time in freezing during pre-CS and CS of cued test {unpaired *t*-test, **p = 0.003 and ***p < 0.001 for **(G)**, *p* = 0.347 and *p* = 0.673 for **(M)**}.

**FIGURE 2:**
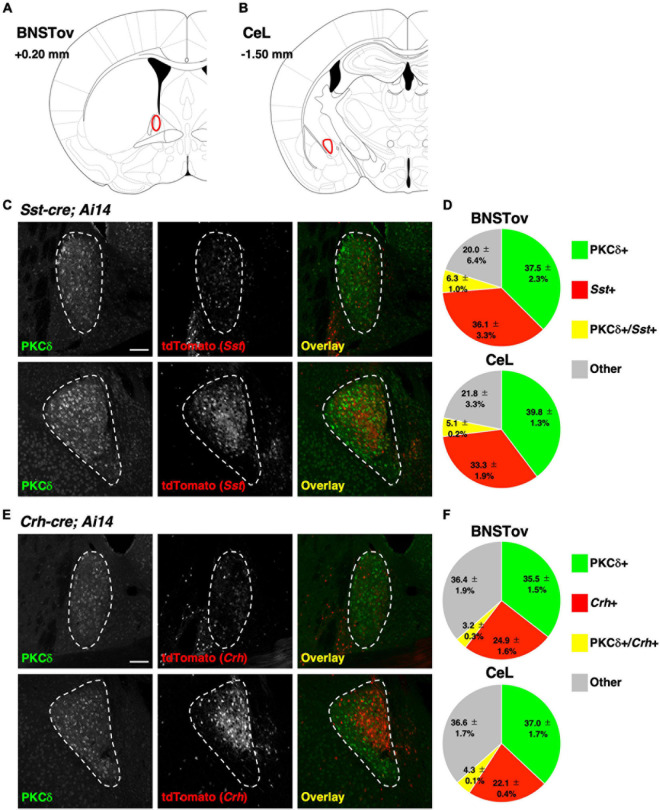
Distribution of genetically defined neuronal populations between the BNSTov and the CeL. **(A,B)** Schematic brain atlas illustrations at the level of the BNSTov **(A)** and the CeL **(B)**. The values indicate anterior–posterior distances from bregma. **(C,E)** Representative images of the BNSTov (upper) and the CeL (lower) from *Sst-cre; Ai14* **(C)** and *Crh-cre; Ai14* **(E)** mouse brains immunolabeled against PKCδ. White enclosing dashed lines indicate the boundaries of the BNSTov or the CeL. Scale bars, 100 μm. **(D,F)** Pie charts showing the proportion of the labeled cells corresponding to (**C,E**; data from 3 mice each). The distribution of genetically defined neuronal populations had no significant difference between the BNSTov and the CeL {Chi-square test, *p* = 0.961 for **(D)**, *p* = 0.964 for **(F)**}.

**FIGURE 3:**
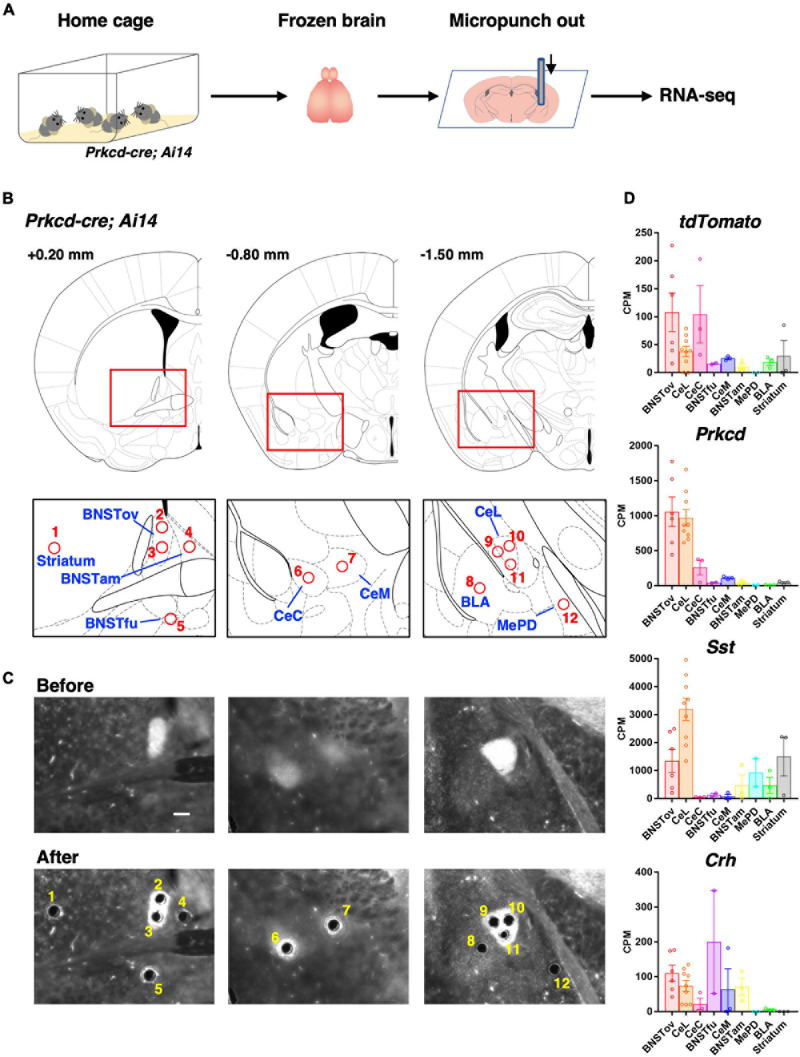
Subnuclei-targeted microtissue collection for RNA-seq. **(A)** Schematic diagram of tissue collection and RNA-seq. **(B)** Schematic brain atlas illustrations of 12 collection points from nine brain regions (1, striatum; 2, 3, BNSTov; 4, BNSTam; 5, BNSTfu; 6, CeC; 7, CeM; 8, BLA; 9–11, CeL; and 12, MePD). Numbers indicate anterior-posterior distances from bregma. **(C)** Representative images of the tdTomato expression of *Prkcd-cre; Ai14* mouse brain sections on polyphenylene sulfide films before (upper) and after (lower) punch out corresponding to **(B)**. Scale bar, 200 μm. **(D)** Expression levels of *tdTomato* and CeL marker genes in nine brain regions represented by counts per million (CPM) values.

### Extraction of mRNA and Preparation of cDNA Library

**FIGURE 4:**
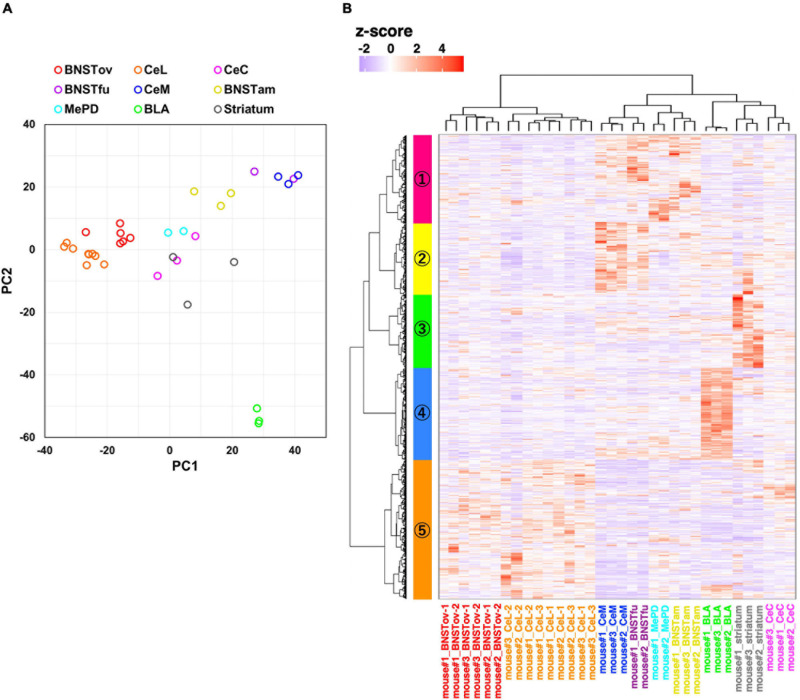
Gene expression profiling from amygdala-related subnuclei. **(A)** PCA using normalized RNA-seq data of 2,800 inter-subnuclei differentially expressed genes (DEGs^subnuc^). **(B)** Hierarchical clustering of relative gene expression of 2,800 DEGs^subnuc^. The horizontal and vertical axes represent each sample and DEGs, respectively. The color scale reflects *z*-score of CPM values.

RNA-seq library construction was performed as previously described (Yamazaki et al., 2020). In brief, microtissue sections were collected by removing the ethanol in PCR tubes using a vacuum evaporator and then lysed in 5.3 μl of cell lysis mixture [PKD buffer (QIAGEN): Proteinase K (QIAGEN) = 16:1] at 56°C for 1 h, and the poly(A) RNA was purified with the oligo dT magnetic beads (61005; Thermo Fisher Scientific) according to the instruction manual. The total amounts of purified mRNA were directly processed according to the Smart-seq2 protocol (Picelli et al., 2014). PCR products were purified with 0.8× volume of AMPure XP beads (A63880; Beckman Coulter). For the comparative experiments of gene expression profiles of the subnuclei (Figures 3, 4), one of three BNSTfu and one of three medial amygdala (MePD) samples were excluded from the sequencing results because their library quality was low, and over 70% of the reads could not be mapped.

### RNA-Seq and Data Analysis

**FIGURE 5:**
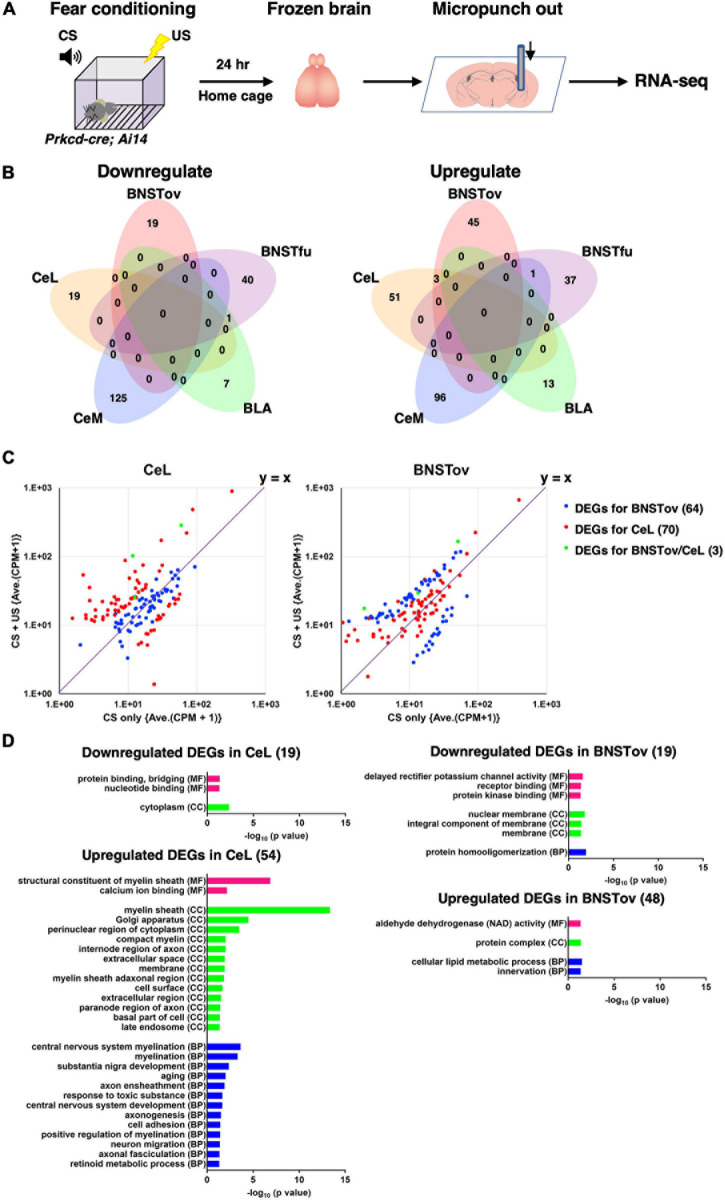
Gene expression changes after fear conditioning. **(A)** Schematic diagram of tissue collection and RNA-seq from mice after fear conditioning. **(B)** Venn diagrams showing the overlap of DEGs in CS-only (control) vs. CS–US (DEGs^fear conditioning^) between five amygdala subnuclei. **(C)** Scatterplots representing the FCs of averaged CPM between CS-only vs. CS–US of DEGs^fear conditioning^ for the BNSTov (blue), the CeL (red), and both (green). The values in parentheses indicate the number of DEGs. **(D)** GO analysis for the downregulated or upregulated DEGs^fear conditioning^ of the BNSTov or the CeL using DAVID Bioinformatics Resources ver. 6.8. Significantly enriched GO terms (*p* < 0.05) in molecular function (MF), cellular component (CC), and biological process (BP) were presented. The values in parentheses indicate the number of DEGs.

Sequencing and data analysis were conducted as previously described (Yoda et al., 2017). The amplified cDNA (0.25 ng) was used for the preparation of the sequencing library, using the Nextera XT DNA library prep kit (Illumina). The libraries were sequenced with 75-bp paired-end read on an Illumina Miseq, as shown in Figure 3, and 150-bp paired-end read on an Illumina Hiseq, as shown in Figure 5. Adapter sequences were trimmed off from the Raw data (raw reads) of fastq format by flexbar (ver. 3.4.0). The resulting reads were aligned to the Ensembl mouse reference genome (GRCm38 ver. 92) by hisat (ver. 2.1.0) with the default parameters. The number of reads assigned to genes was calculated using featureCounts (ver. 1.6.4). To normalize for the differences in sequencing depth across samples, protein coding gene counts were rescaled to counts per million (CPM) by Trimmed Mean of *M*-values normalization from edgeR. Low-expression genes with an average fewer than 10 CPM in all subnuclei were excluded from analysis. Differential gene expression analysis was performed using the R/Bioconductor package edgeR (ver. 3.32.0). Inter-subnuclei differentially expressed genes (DEGs^subnuc^) for Figure 4 were defined as false discovery rate (FDR) < 0.01 and maximum inter-subnuclei fold change (FC; averaged CPM value of the most expressed subnucleus/averaged CPM value of the least expressed subnucleus) > 2. DEGs between CS-only (as control) and CS–US exposed mice (DEGs^fear conditioning^) for Figure 5 were defined as *p*-value < 0.01 and | log_2_ FC (CS-only vs. CS–US)| > 1. Principal component analysis (PCA) was performed using the R (ver. 4.0.2) package prcomp with option scale = TRUE. Hierarchical clustering was performed using the R package hclust with option method = ward.D2. Gene ontology (GO) enrichment analysis was performed using DAVID Bioinformatics Resources (ver. 6.8)^1^.

### Statistics

All values were expressed as mean ± s.e.m. Chi-square tests, unpaired two-tailed *t*-tests, and two-way RM analysis of variance followed by Holm–Sidak’s multiple comparison tests were performed using the GraphPad Prism software (ver. 7).

## Results

### Inhibiting Neurotransmission of CeL^PKCδ+^ Neurons Attenuated Fear Learning, Whereas That of the BNSTov^PKCδ+^ Affected Anxiety-Like Behavior

There are few studies focusing on the functional comparison between the CeA and the BNST and their functional differences are still considered to be controversial (Lebow and Chen, 2016). To compare the contribution of their subnuclei in the regulation of fear and anxiety, PKCδ+ neuronal transmission in the CeL or the BNSTov was constitutively inhibited by expression of tetanus toxin light chain (TeNT) to disturb proper local circuit function and investigated its impact on the behaviors related to two distinct negative emotion, fear and anxiety, under the same experimental conditions.

To inhibit the neurotransmission of the PKCδ+ neurons either in the CeL (CeL^PKCδ^^+^) or the BNSTov (BNSTov^PKCδ^^+^), cre-dependent TeNT-expressing AAV were bilaterally injected into each subnucleus of *Prkcd-cre* mice. 4 weeks later, a series of behavioral experiments were conducted (Figures 1A,B,H). The experiments included, OF test, EPM test, and contextual and cued fear conditioning test. The tests were conducted to assess spontaneous activity, anxiety-related behavior, and fear-related behavior, respectively. Neither CeL^PKCδ^^+^- nor BNSTov^PKCδ^^+^-silencing mice showed significant changes in spontaneous locomotor activity in the OF test (Figures 1C,I). CeL^PKCδ^^+^-silencing mice showed declined fear learning during the conditioning (Figure 1E) and reduced freezing ratios in the contextual and cued tests (Figures 1F,G), suggesting CeL^PKCδ^^+^ inhibition induced fear-learning deficit in consistent with the previous literature (Yu et al., 2017). In the cued tests, CeL^PKCδ^^+^-silencing mice also showed significantly reduced freezing during the pre-CS period (Figure 1G; control: 10.13 ± 2.37%, *n* = 13, TeNT: 1.91 ± 0.89%, *n* = 14, *p* = 0.003). The non-negligible freezing exhibited by the control mice during the pre-CS period (10.13 ± 2.37%) suggested that the learned fear was generalized to some extent to the context used in the cued test under our experimental conditions as reported previously (Huckleberry et al., 2016). As the fear generalization has been regarded as a byproduct of conditioned learning and could be affected by learning processes (Dunsmoor and Paz, 2015; Asok et al., 2019), the reduced freezing during the pre-CS period in the CeL^PKCδ^^+^-silencing mice may be partly explained by the attenuated generalized fear attributable to the fear-learning deficit. Conversely, there were no significant differences in anxiety-like behavior in the EPM (Figure 1D). In contrast, BNSTov^PKCδ^^+^-silencing mice spent a significantly longer time in the open-arm of the EPM, indicating that BNSTov^PKCδ^^+^ inhibition attenuated anxiety-like behaviors, while they showed no difference in the contextual and cued fear conditioning tests (Figures 1J–M). The anxiety-like behavior assessed by OF test (e.g., time spent in center area) did not show significant differences between groups (data not shown), suggesting BNSTov^PKCδ^^+^-silencing induced the context-dependent effects on anxiety-like behavior which was discussed in the literature (Holmes et al., 2003). These results, obtained under exactly the same conditions, showed a double dissociation of CeL^PKCδ+^ vs. BNSTov^PKCδ+^ neurons’ involvement in negative emotional behaviors, with the former contributing much more in fear learning while the latter doing so in anxiety expression.

### The CeL and the BNSTov Showed Similar Composition of Genetically Identified Cell Types

We next investigated the differences in the composition of major neuronal cell types in the CeL vs. the BNSTov which may account for the dissociable influence on the behavioral regulation mediated by PKCδ+ neurons in these subnuclei. The CeL circuitry has been well studied as an intranuclear reciprocal inhibitory circuit that gates emotional behavioral expression between genetically distinct neuronal populations, PKCδ+ and PKCδ-negative (PKCδ-) neurons (represented by Sst+ neurons or Crh+ neurons), and it has been reported that three cell types cover majority of neurons in the CeL (Ciocchi et al., 2010; Haubensak et al., 2010; Li et al., 2013; Fadok et al., 2017; Kim et al., 2017; Sanford et al., 2017; McCullough et al., 2018; Hartley et al., 2019). Recent studies have reported that these molecules are also expressed in the lateral BNST, especially in the BNSTov subnucleus (Wang et al., 2019, 2020). When the cre-reporter line, *Ai14*, was crossed with a CeA cre-driver line (*Prkcd-cre*, *Sst-cre*, or *Crh-cre*), a high-level expression of the reporter gene, tdTomato, was consistently observed in the CeL and the BNSTov (Supplementary Figure 1). To compare the distribution of these genetically defined neuronal populations between the CeL and the BNSTov, immunohistochemical analysis was performed for PKCδ on *Sst-cre; Ai14* or *Crh-cre; Ai14* mice (Figure 2). Counting of the number of each cell type indicated that the fraction of PKCδ+ neurons and *Sst*+ or *Crh*+ neurons in the BNSTov was considerably similar to the CeL (Figures 2D,F). Furthermore, BNSTov^PKCδ^^+^ or CeL^PKCδ^^+^ rarely overlapped with the genetically labeled *Sst*+ or *Crh*+ neurons (Figures 2D,F), as was reported in previous studies (Kim et al., 2017; McCullough et al., 2018). These results showed quite similar composition of three major cell types between the CeL and the BNSTov, indicating that the distinctive functional output did not result from the difference in cell composition.

### Fluorescence-Assisted Subnuclei-Targeted Gene Expression Analysis Revealed Similar Constitutive Molecular Profiles of the CeL and the BNSTov

We next compared the molecular constituents of the CeL, the BNSTov, and other related regions in an unbiased way. Subnuclei-targeted gene expression analysis was performed using RNA-seq on microsamples produced by a microdissection punching system (Figure 3A; Yoda et al., 2017; Yamazaki et al., 2020). To identify each extended amygdala subnuclei and other related subregions, *Prkcd-cre; Ai14* mouse brains were used, in which the cell bodies, dendrites, and axons of the PKCδ+ neurons were labeled with tdTomoto fluorescence. The subnuclei in the central extended amygdala, such as the BNSTov, the CeL, and the capsular subdivision of the CeA (CeC), where the cell bodies of PKCδ+ neurons are density located, were marked with the prominent tdTomato fluorescence (Supplementary Figure 1B). We also found that the regions containing dense-projecting axons were labeled moderately, which was useful in the precise identification of CeL^PKCδ^^+^- and/or BNSTov^PKCδ^^+^-projecting regions, such as the medial subdivision of the CeA (CeM) and the fusiform nucleus of the BNST (BNSTfu; Supplementary Figure 1B; Dong et al., 2001b; Daniel and Rainnie, 2016; Wang et al., 2019). Furthermore, we determined the sampling position in the neighboring nuclei—the striatum, anteromedial subdivision of the BNST (BNSTam), basolateral amygdala (BLA), and posterodorsal subdivision of the MePD—with reference to the fluorescent labeling. Thus, the locations of the microsamples for eight amygdala-related subnuclei and neighboring striatum were determined. The number of sampling locations in each region was as follows: CeL, 3; BNSTov, 2; BNSTam, 1; BNSTfu, 1; CeC, 1; CeM, 1; BLA, 1; MePD, 1; and striatum, 1. Microsamples were collected with a 110-μm-inner diameter punching needle while observing tdTomato (Figures 3A–C). These samples were then prepared for gene expression analysis using RNA-seq. Because the expression levels of *tdTomato* were expected to be high in the BNSTov, CeL, and CeC, where the cell bodies are labeled, high expressions of *tdTomato* were checked in these samples (Figure 3D). In addition to *Prkcd*, *Sst*, and *Crh* being highly expressed in the CeL, other known markers were observed to have similarly high levels of expression, which included neurotensin (*Nts*), tachykinin 2 (*Tac2*), and preprodynorphin (*Pdyn*; Figure 3D and Supplementary Figure 2).

Following the confirmation of the accuracy of the system, to evaluate the similarity of the molecular profiles between subnuclei, 2,800 inter-subnuclei DEGs were extracted among nine subnuclei (DEGs^subnuc^; defined as FDR < 0.01 and maximum inter-subnuclei FC > 2). PCA was then conducted on these genes. Consistent with the hypothesis that the CeL and the BNSTov have similar molecular profiles, these samples (BNSTov, red circles; CeL, orange circles) were plotted adjacent to each other (Figure 4A). Interestingly, the BNSTfu samples (violet circles) were plotted adjacent to CeM samples (Figure 4A, blue circles). Furthermore, hierarchical clustering analysis was carried out based on the same 2,800 DEGs^subnuc^ (Figure 4B). The clustering of the 34 microdissected samples revealed that the CeL and the BNSTov samples were classified under the same cluster (Figure 4B, *X*-axis). The number of samples (animals) in each region was as follows: BNSTov, 6 (3); CeL, 9 (3); CeC, 3 (3); BNSTfu, 2 (2); CeM, 3 (3); BNSTam, 3 (3); MePD, 2 (2); BLA, 3 (3), and striatum, 3 (3). Furthermore, 2,800 DEGs^subnuc^ were divided into five clusters (Figure 4B, *Y*-axis and Supplementary Table 1). The cluster #5 (colored orange) included 837 genes with high expression levels in both the CeL and the BNSTov (Supplementary Table 1). Indeed, the gene set included genes known to be highly expressed in the CeL and/or the BNSTov (e.g., *Prkcd*, *Sst*, *Nts*, *Tac2*, *Pdyn*, and *Camk1g*; Takemoto-Kimura et al., 2003) and also genes whose predominant expression in the CeL and/or the BNSTov have not been reported thus far (e.g., *Ncdn*, *WSM1*, *Ptprn*, *Fkbp1a*, and *Dock10*, Supplementary Figure 2). Taken together, the data revealed that the CeL and the BNSTov shared similar gene expression profiles at the basal level.

### The CeL and the BNSTov Displayed Distinctive Gene Expression Responses to Fear-Related Stimuli

The extended amygdala is known to alter its circuit activity and function in the face of stress to adapt to environmental changes (Herman et al., 2003; Zhang et al., 2021). Although both the CeL and the BNSTov are implicated in the regulation of fear processing (Goosens and Maren, 2001; Sullivan et al., 2004), our results indicated the distinctive extent of contribution to the fear learning between the subnuclei and suggested the existence of molecular programs underlying subnuclear specific behavioral regulation. In order to examine whether each subnucleus differentially responds in gene expression in the face of fear-related stimulus, the fluorescence-assisted subnuclei-targeted gene expression analysis was conducted, where animals experienced CS–US exposure in a classical fear conditioning.

Twenty-four hours after the CS–US exposure, which is the time point at which long-term fear memory is known to be established (Figure 1), the microdissected samples were collected from five amygdala-related subnuclei of *Prkcd-cre; Ai14* mice using the same strategy as Figure 3, followed by the RNA-seq (Figure 5A). The number of samples (animals) in each region was as follows: BNSTov, 6 (3); BNSTfu, 3 (3); CeM, 3 (3); BLA, 3 (3); and CeL, 9 (3). First, we confirmed that three marker gene (*Prkcd*, *Sst*, and *Crh*) expression levels in the CeL and the BNSTov showed no significant differences in CPM values between CS-only (as control) and CS–US exposed mice, indicating that there were no apparent cell-type compositional changes in CS–US exposed mice (data not shown). Data analysis revealed 19 down-regulated and 54 up-regulated genes in the CeL and 19 down-regulated and 48 up-regulated genes in the BNSTov as DEGs between CS-only and CS–US exposed mice (DEGs^fear conditioning^; defined as *p*-value < 0.01 and | log_2_ FC| > 1; Supplementary Table 2). Only three genes were identified as up-regulated DEGs both in the BNSTov and the CeL (Figure 5B), showing specific responses in each subnucleus. To visualize the tendency and range of gene expression changes in the BNSTov and the CeL, the scatterplots of DEGs for the BNSTov and the CeL were created (Figure 5C). Most BNSTov-specific DEGs (blue) were observed near the symmetry line (*y* = *x*) on the plot for the CeL (Figure 5C, left), and *vice versa* for CeL-specific DEGs (red) on the plot for the BNSTov (Figure 5C, right). This indicated that the trend of gene expression alteration between the two subnuclei substantially differed quantitatively. Indeed, the CeL showed a larger number of markedly up-regulated genes than the BNSTov (16/54 genes in the CeL and 8/48 genes in the BNSTov were up-regulated more than five times). Furthermore, hierarchical clustering analysis of gene expression FCs in CS–US exposed mice showed that the CeL and the BNSTov were separated in subtrees on opposite sides (Supplementary Figure 3).

Next, GO annotation enrichment analyses were performed to describe the functional properties of DEG sets identified in the CeL and the BNSTov, respectively, and no GO term overlapped between the CeL and the BNSTov (Figure 5D and Supplementary Table 3). Notably, for the up-regulated DEGs in the CeL, multiple myelin-related GO terms were listed, raising the possibility that CS–US-induced fear learning promoted myelination remodeling in the CeL. Together, these results led to a conclusion that the gene expression responses in the CeL and the BNSTov, when facing fear-related stimuli, significantly differ both quantitatively and qualitatively. The distinctive gene expression responses may reflect the subnuclear specific activities in response to fear-related stimuli, which possibly lead to the functional differences between these subnuclei.

## Discussion

Recent advances in tools and methodologies, such as genetically targeted *in vivo* imaging and circuit manipulation, have expanded our understanding of the CeA circuit function within the extended amygdala (Janak and Tye, 2015; Gafford and Ressler, 2016; Babaev et al., 2018; Fadok et al., 2018). However, the elucidation of the BNST circuitry function remains less understood because of the complexities of the intra- and inter-subnuclear circuits, which are composed of nearly 20 subnuclei (Fox et al., 2015; Lebow and Chen, 2016). In the present study, we found that the neurotransmission inhibition of PKCδ+ neurons—a major cell type of both the CeL and the BNSTov—induced subnuclei-specific double dissociation on fear and anxiety regulation. Consistently, these subnuclei exhibited distinctive gene expression in response to fear-related stimuli, despite their similarity in constitutive gene expression profiles.

To perform subnuclei-targeted gene expression analysis, tissue microdissection punching systems were operated using a needle with an inner diameter of 110 μm from 20-μm-thick brain sections. These microtissues were estimated to contain between 30 and 50 cells, with the suitable size then determined for capturing the special molecular properties of small regions (Yoda et al., 2017). Hierarchical clustering analyses showed high reproducibility, since each subnuclei was classified under the same subtree. Molecular profile similarities were found between the CeL and the BNSTov, and the CeM and the BNSTfu. Notably, these subnuclei make up the central extended amygdala, which was originally conceptualized based on developmental comparisons and later distinguished as cell groups on the basis of the similarities of its neurochemical and anatomical properties (Alheid and Heimer, 1988; Johnston, 1923; Alheid et al., 1998). The present results can be said to confirm the similar molecular constituents of these regions by presenting high-precision comprehensive gene expression data for individual subnucleus. Furthermore, the present study not only confirmed the previous studies but also picked up novel genes enriched in the CeL and the BNSTov that belong to the same cluster containing *Prkcd*, such as *Ncdn*, *WSM1*, *Ptprn*, *Fkbp1a*, and *Dock10* (colored orange in Figure 4B). These genes may be useful for genetic dissection as novel cell populations that together compose subnuclei, which may constitute important functional units.

In sharp contrast to the similarity in molecular profiles observed between these subnuclei at the basal state, CeL-specific gene expression responses were found to be induced by CS–US exposure. This may likely be accounted for by CeL-specific activities when animals were exposed to fear-related stimuli, although the direct association between the identified DEGs in the CeL and the activity of CeL^PKCδ+^ neurons which affects fear-learning is unclear and needs to be validated. Interestingly, myelination-related pathways were enriched in the DEGs within the CeL by CS–US exposure. Although further studies are required to reveal the time course, mechanism, and biological outcome of up-regulated gene expression of myelination-related genes in the CeL, it is noteworthy that, in recent studies, neuronal activity-induced changes in myelination have been considered one of the important factors of neural plasticity (Xin and Chan, 2020). Experience-driven myelination is induced in a circuit activity-dependent manner and then strengthens specific neural circuits through facilitating action-potential transmissions (Fields, 2015; de Faria et al., 2019). Actually, in the medial prefrontal cortex, it has recently been reported that new myelin formation is induced by fear learning and is required for preservation of fear memory (Pan et al., 2020). It is possible that fear-inducing stimuli promote myelination and strengthened internuclear fear-related circuits, such as BLA-CeL and parabrachial nucleus-CeL circuits (Janak and Tye, 2015; Palmiter, 2018).

In addition, the results of the present study showed that the extent of involvement in the regulation of fear or anxiety processing may differ between the CeL^PKCδ^^+^ and the BNSTov^PKCδ^^+^ neurons. Namely, the former makes a greater contribution toward fear learning, while the latter does toward anxiety expression. This is in keeping with the notion suggested in human imaging studies, that the CeA and the BNST may have different degrees of contribution toward the regulation of fear and anxiety (Klumpers et al., 2017; Gorka et al., 2018; Hur et al., 2020). The constitutive cell type composition and gene expression were similar between the CeL and BNSTov, and could not explain the functional differences; hence, we presumed that other features such as input/output circuit differences may be involved. However, further studies are needed to clarify this issue. In addition, since the present circuit manipulation study is limited to PKCδ+ neurons and RNA-seq was performed using micropunch samples containing heterogeneous cell types, further investigation on the functions of other cell-types and the integrated local circuit is required to understand the mechanism underlying the behavioral differences observed in this study.

A previous study using the same mouse line has reported that transient chemogenetic activation of the PKCδ+ neurons of the BNST reduces anxiety (Wang et al., 2020). This result seems to be superficially inconsistent with the present study, as we found that constitutive silencing of PKCδ+ neurons of the BNSTov diminished anxiety behavior, which implies that some BNSTov^PKCδ+^ activity is needed for anxiety expression. Thus, BNST may be composed of mixed populations of either anxiogenic, anxiolytic or dually active cells. The other possibility is that acute activation using a chemogenetic method induces a synchronized local circuit dysfunction, which may show resemblance to outcomes observed with the chronic silencing used in this study. All these studies, nonetheless, would agree with the idea that proper activity of PKCδ+ neurons in the BNSTov is essential for gating anxiety-related behavior expression.

Together, these results suggest that emotional behavior-related information representation is functionally organized distinctly in each subnuclei of the extended amygdala, although individual neuronal cell types appear to be largely conserved based on constitutive gene expression profiles. Subsequent studies on the relationship between the identified gene expression responses and behavioral changes will be needed to understand the subnuclear specific molecular mechanisms underlying the regulation of emotional behaviors. Moreover, future dissection of active ensembles in extended amygdala subnuclei together with the molecular mechanisms underlying their regulation, will pave the way toward better understanding of complex dynamics of information integration within the amygdala during transition between various emotional states.

## Data Availability Statement

All RNA-seq data were deposited in DNA Data Bank of Japan (DDBJ) under the accession numbers DRA012354 and DRA012355. Other datasets generated and/or analyzed during the current study are available from the corresponding author at reasonable request.

## Ethics Statement

All animal experiments were conducted in accordance with the Nagoya University Regulations on Animal Care and Use in Research and were approved by the Institutional Animal Care and Use Committee, Nagoya University (approval number R210154).

## Author Contributions

SU and ST-K designed the project and wrote the first draft of the manuscript. SU, MF, ManK, TF, HK, SH, KI, MasK, HB, and ST-K contributed to histological analyses, behavioral experiments, and interpretation of results. SU, MH, KA, KT, HM, and HT performed RNA-seq and analyzed the sequence data. All authors read and approved the final manuscript.

## Conflict of Interest

The authors declare that the research was conducted in the absence of any commercial or financial relationships that could be construed as a potential conflict of interest.

## Publisher’s Note

All claims expressed in this article are solely those of the authors and do not necessarily represent those of their affiliated organizations, or those of the publisher, the editors and the reviewers. Any product that may be evaluated in this article, or claim that may be made by its manufacturer, is not guaranteed or endorsed by the publisher.

## Footnotes

1
https://david.ncifcrf.gov/home.jsp
