# Supplementary material for: Distinctive Regulation of Emotional Behaviors and Fear-Related Gene Expression Responses in Two Extended Amygdala Subnuclei With Similar Molecular Profiles
Source: Front Mol Neurosci. 2021 Sep 3;14:741895. doi: 10.3389/fnmol.2021.741895 (PMC8446640; doi:10.3389/fnmol.2021.741895)
Supplement: Supplementary file 4 [file Image_1.pdf]

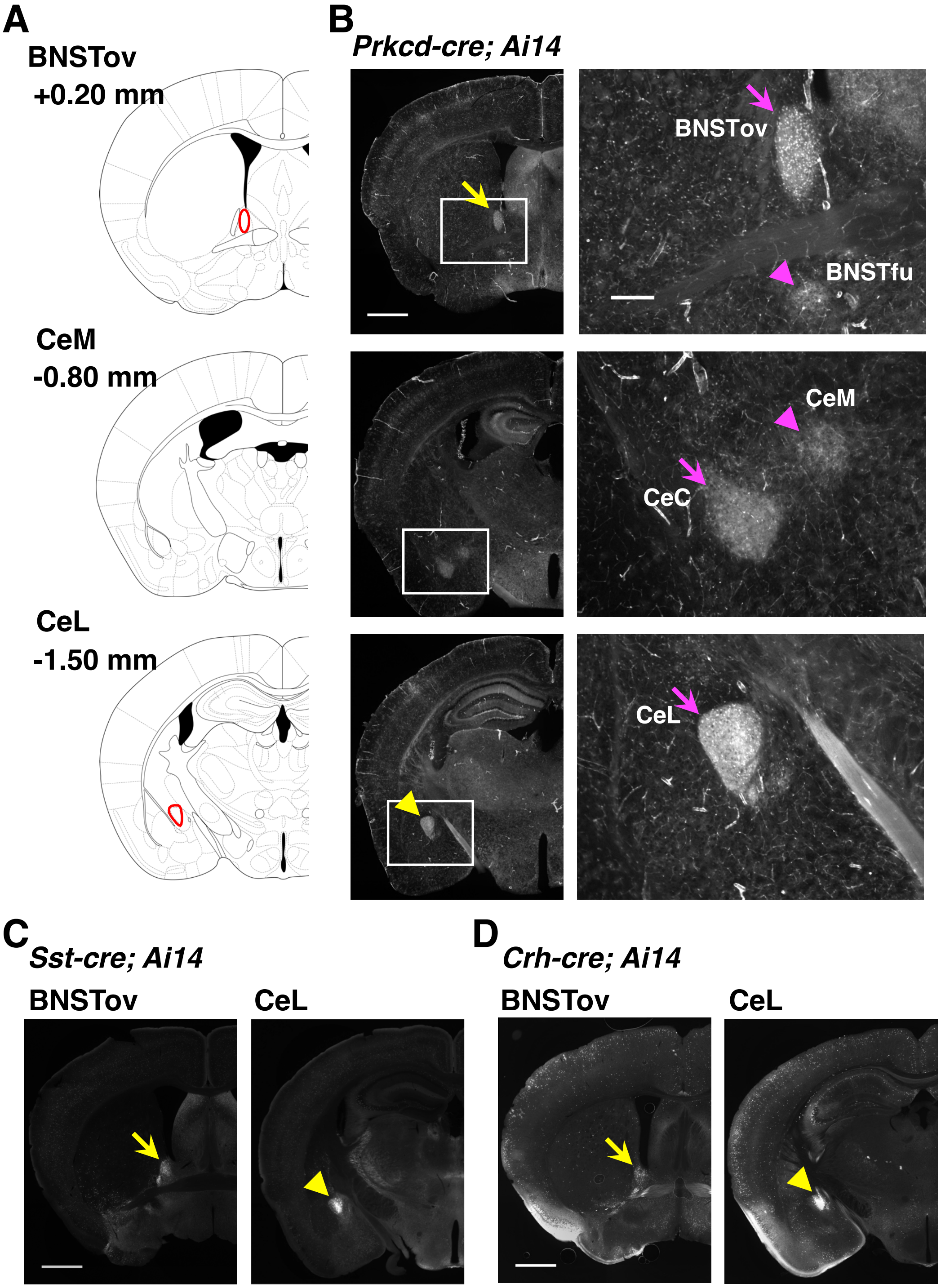

Supplementary Figure 1. Distribution of genetically defined neuronal populations. **(A)** Schematic brain atlas illustrations at the level of the BNSTov, CeM and CeL. The values indicate anterior-posterior distances from bregma. **(B)** Representative coronal section images of tdTomato expression of *Prkcd-cre; Ai14* mouse brains corresponding to (A) (left) and higher magnification images of white boxed (right). Scale bars, 1 mm and 200 μm. Yellow arrow and arrowhead indicate the BNSTov and the CeL, respectively. Pink arrows and arrowheads indicate subnuclei with PKCδ+ cell bodies and axonal terminals, respectively. Scale bar, 1 mm. **(C, D)** Representative coronal section images of the tdTomato expression of *Sst-cre; Ai14* (C) and *Crh-cre; Ai14* (D) mouse brains. Yellow arrows and arrowheads indicate the BNSTov and the CeL, respectively. Scale bars, 1 mm.
