# Supplementary material for: Distinctive Regulation of Emotional Behaviors and Fear-Related Gene Expression Responses in Two Extended Amygdala Subnuclei With Similar Molecular Profiles
Source: Front Mol Neurosci. 2021 Sep 3;14:741895. doi: 10.3389/fnmol.2021.741895 (PMC8446640; doi:10.3389/fnmol.2021.741895)
Supplement: Supplementary file 5 [file Image_2.pdf]

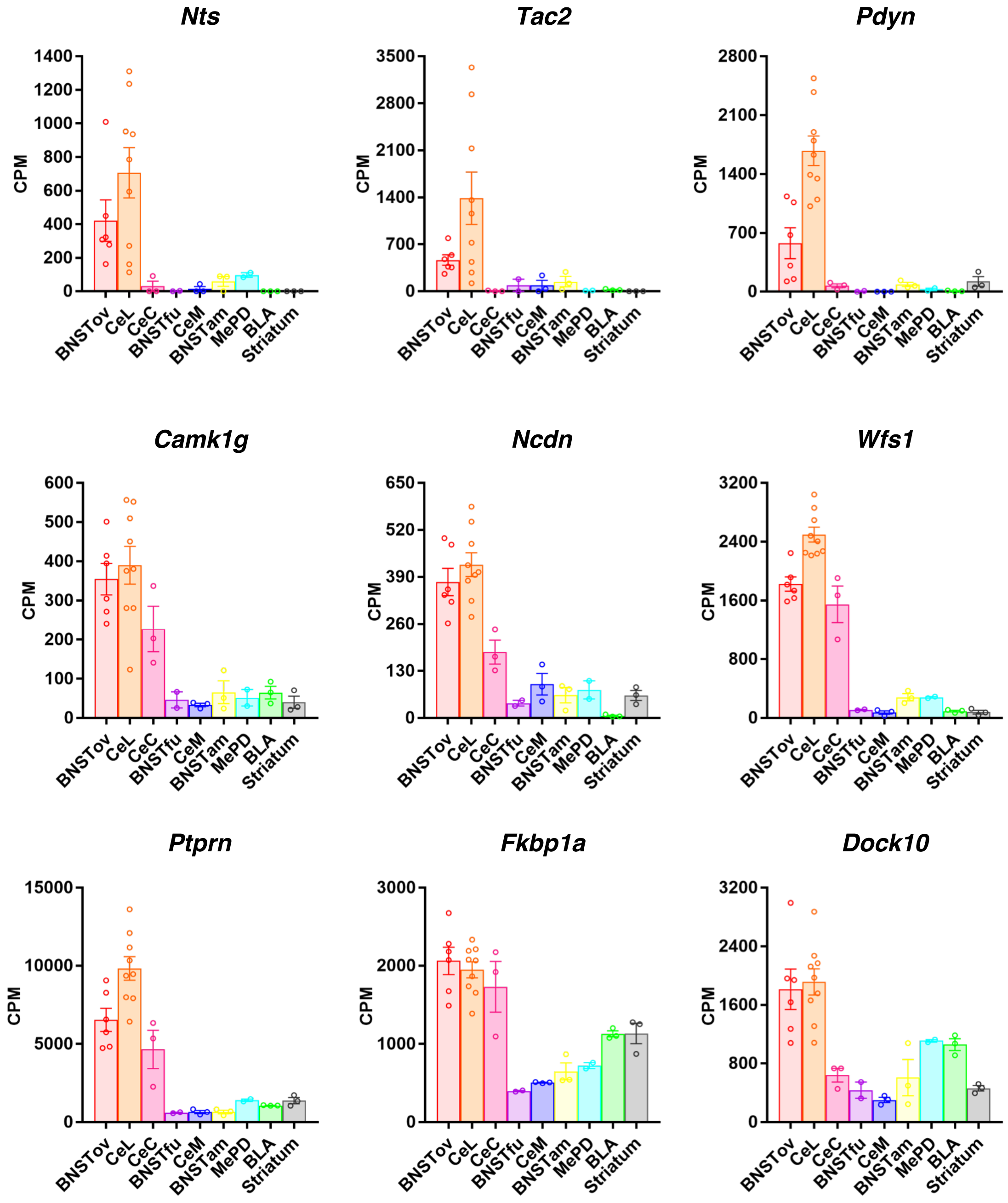

Supplementary Figure 2. Subnuclei-specific gene expression profiling using RNA-seq. Expression levels of known CeL marker genes (*Nts*, *Tac2*, *Pdyn*, and *Camk1g*) and representative genes belonging to cluster #5 (colored orange) of Figure 4B (*Ncdn*, *Wfs1*, *Ptprn*, *Fkbp1a*, and *Dock10*) in nine brain regions represented by CPM values.
