# Supplementary material for: Distinctive Regulation of Emotional Behaviors and Fear-Related Gene Expression Responses in Two Extended Amygdala Subnuclei With Similar Molecular Profiles
Source: Front Mol Neurosci. 2021 Sep 3;14:741895. doi: 10.3389/fnmol.2021.741895 (PMC8446640; doi:10.3389/fnmol.2021.741895)
Supplement: Supplementary file 6 [file Image_3.pdf]

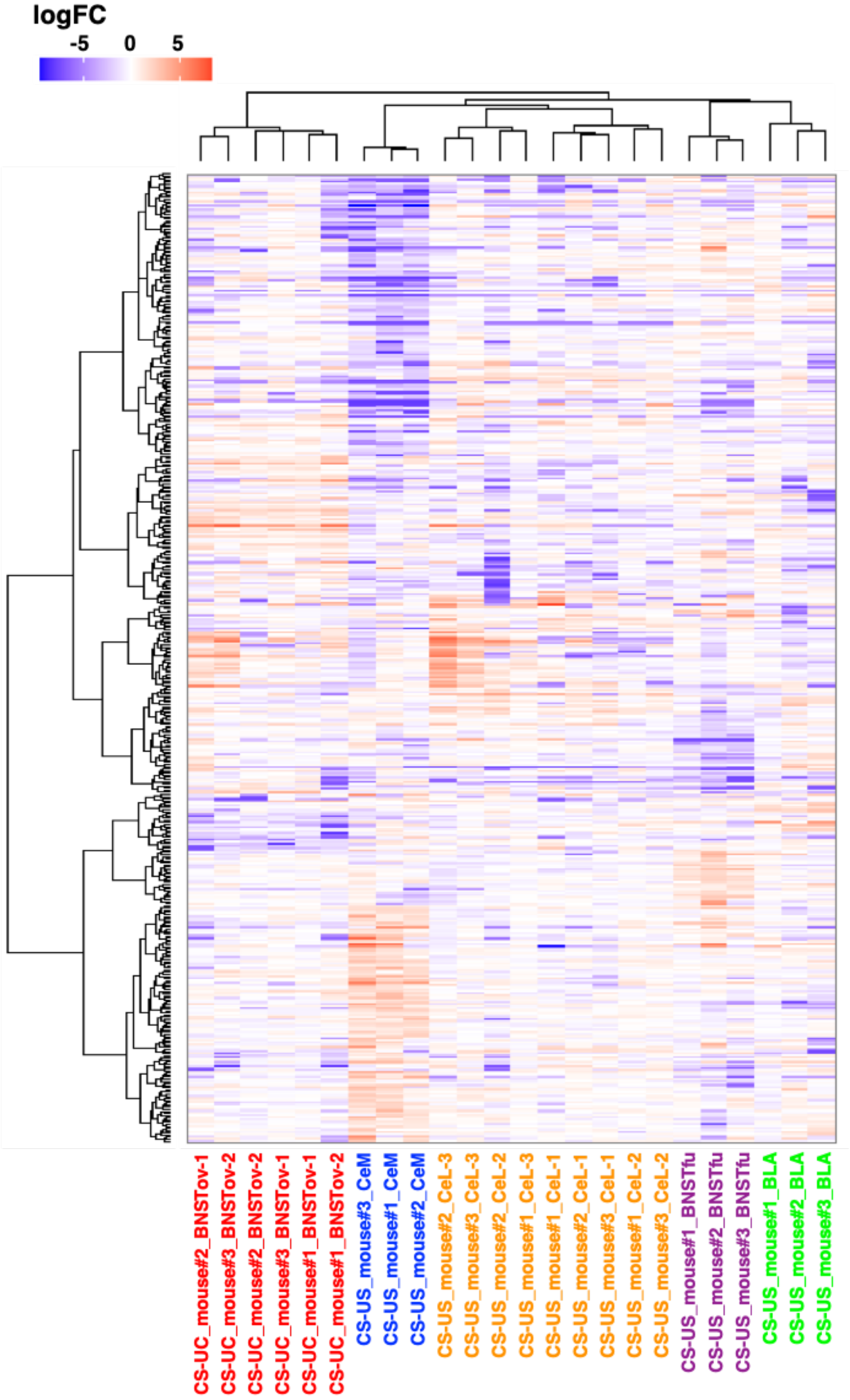

Supplementary Figure 3. Hierarchical clustering of gene expression changes after fear conditioning. Hierarchical clustering of FCs in gene expression of 457 DEGs$^{\text{fear conditioning}}$. The horizontal and vertical axes represent each sample and DEGs, respectively. The color scale reflects $\log_2$ FCs calculated by dividing the averaged CPM values of CS-only control.
